# Supplementary material for: Induction and Suspension Culture of Panax japonicus Callus Tissue for the Production of Secondary Metabolic Active Substances
Source: Plants (Basel). 2024 Sep 4;13(17):2480. doi: 10.3390/plants13172480 (PMC11396918; doi:10.3390/plants13172480)
Supplement: Supplementary file 1 [file plants-13-02480-s001.zip › plants-3129180-supplementary.pdf]

Table S1. Effects of different disinfection methods on the growth status of the roots of *Panax japonicus*

Scheme	the concentration of NaClO/%	Disinfection time /min	Contamination rates /%	Mortality /%	Survival rate /%
1	3	8	100.00	0	0
2	3	10	75.00	0	25.00
3	3	12	65.00	0	35.00
4	3	15	50.00	0	30.00
5	3	18	70.00	0	25.00
6	3	20	85.00	0	15.00
7	5	15	20.00	0	80.00
8	5	18	30.00	0	70.00
9	5	20	35.00	0	65.00
10	10	15	5.00	0	95.00
11	10	18	15.00	10.00	75.00
12	10	20	20.00	15.00	65.00

Table S2. Effects of different hormone levels on callus induction of *Panax japonicus* and Analysis of Range and Variance

Treatment	NAA（μM）	6-BA（μM）	Callus induction rate（%）
1	1（0）	1（0）	0
2	1	2（6.66）	22.00
3	1	3（13.32）	31.83
4	1	4（26.64）	30.50
5	2（8.06）	1	34.83
6	2	2	66.67
7	2	3	71.83
8	2	4	57.50
9	3（16.11）	1	66.00
10	3	2	79.17
11	3	3	88.17
12	3	4	57.50
13	4（32.22）	1	50.50
14	4	2	60.17
15	4	3	67.50
16	4	4	24.17
K1	84.33	151.33	—
K2	230.83	228.00	—
K3	290.83	259.33	—
K4	202.33	169.67	—
k1	21.08	37.83	—
k2	57.71	57.00	—
k3	72.71	64.83	—
k4	50.58	42.42	—
R	51.63	27.00	—
F	93.09	28.57	—
P	＜0.01	＜0.01	—

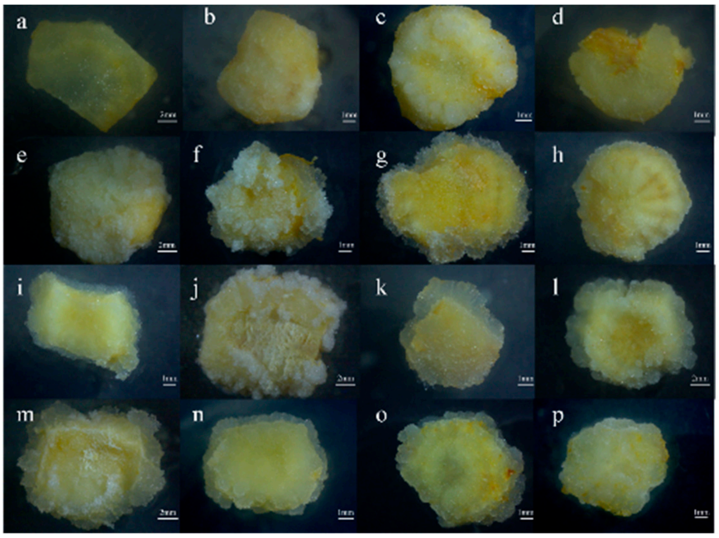

Figure S1. The effect of different hormone levels on the morphology of *Panax japonicus* callus tissue

Note: a-p show the induction of callus tissue in treatments 1-16

(the hormone combinations for each treatment are listed in Table S2)

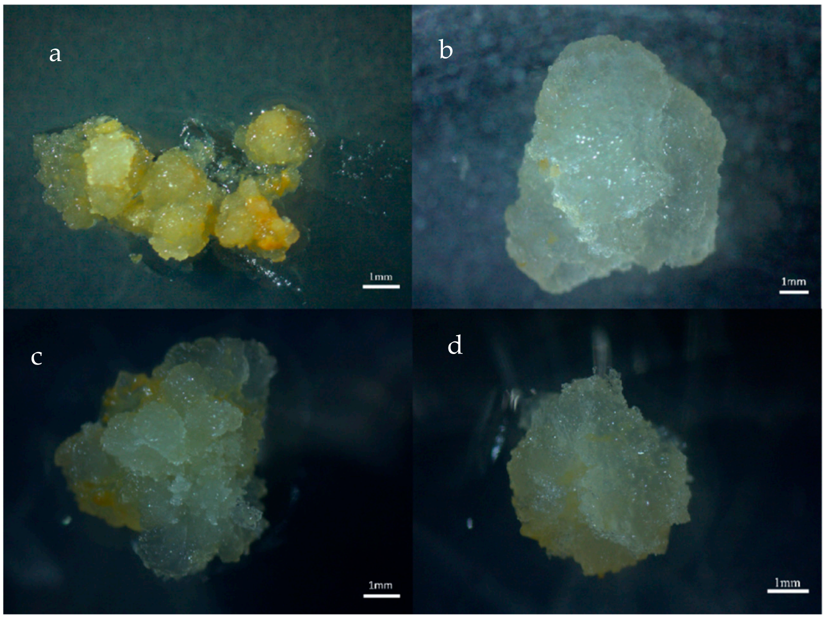

Figure S2. The proliferation and condition of *Panax japonicus* callus on different proliferation medium.

Note: a, the callus is the yellow and dense structure and not agglomerated; b, the callus is white and transparent, the structure is relatively dense; c, the callus is white and transparent, relatively loose structure; d, the callus is White and transparent, loose structure.

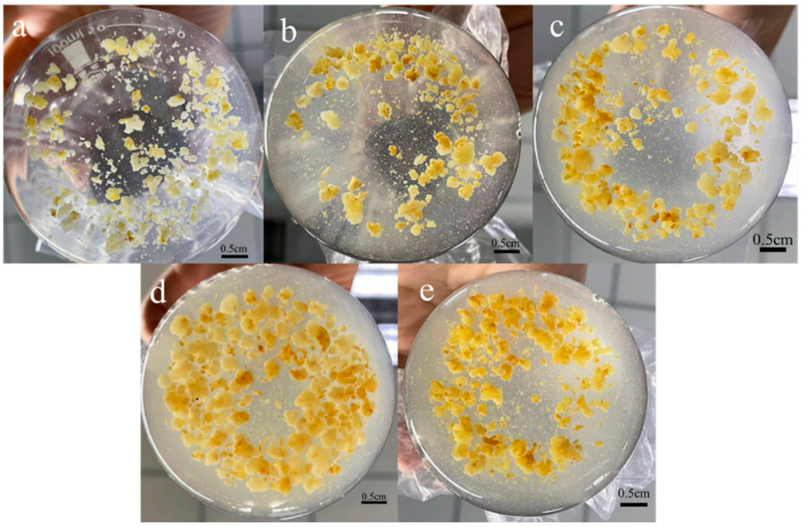

Figure S3. The state of the callus suspension culture of *Panax japonicus*

Note: a is the state of the callus tissue at 0 days of culture; b is the state of the callus tissue at 7 days of culture; c is the state of the callus at 14 days of culture; d is the state of the callus tissue at 21 days of culture; e is the state of the callus tissue at 28 days of culture.
